# See One, Do One, Order One: a study protocol for cluster randomized controlled trial testing three strategies for implementing motivational interviewing on medical inpatient units

**DOI:** 10.1186/s13012-015-0327-9

**Published:** 2015-09-29

**Authors:** Steve Martino, Paula Zimbrean, Ariadna Forray, Joy Kaufman, Paul Desan, Todd A. Olmstead, Ralitza Gueorguieva, Heather Howell, Ashley McCaherty, Kimberly A. Yonkers

**Affiliations:** Psychology Service, VA Connecticut Healthcare System, 950 Campbell Avenue (116B), West Haven, CT 06516 USA; Department of Psychiatry, Yale University School of Medicine, 300 George Street, Suite 901, New Haven, CT 06511 USA; Lyndon B. Johnson School of Public Affairs, The University of Texas at Austin, 2300 Red River St., Stop E2700, Sid Richardson Hall, Unit 3, Austin, TX 78712 USA; Seton/UT Clinical Research Institute, 1400 North IH 35, Austin, TX 78701 USA; Department of Biostatistics, Yale University School of Medicine, 60 College Street, New Haven, CT 06510 USA

**Keywords:** Primary care integration, Implementation strategies, Motivational interviewing

## Abstract

**Background:**

General medical hospitals provide care for a disproportionate share of patients who abuse or are dependent upon substances. This group is among the most costly to treat and has the poorest medical and addiction recovery outcomes. Hospitalization provides a unique opportunity to identify and motivate patients to address their substance use problems in that patients are accessible, have time for an intervention, and are often admitted for complications related to substance use that renders hospitalization a “teachable moment.”

**Methods/Design:**

This randomized controlled trial will examine the effectiveness of three different strategies for integrating motivational interviewing (MI) into the practice of providers working within a general medical inpatient hospitalist service: (1) a continuing medical education workshop that provides background and “shows” providers how to conduct MI (See One); (2) an apprenticeship model involving workshop training plus live supervision of bedside practice (Do One); and (3) ordering MI from the psychiatry consultation-liaison (CL) service after learning about it in a workshop (Order One). Thirty providers (physicians, physician assistants, nurses) will be randomized to conditions and then assessed for their provision of MI to 40 study-eligible inpatients. The primary aims of the study are to assess (1) the utilization of MI in each condition; (2) the integrity of MI when providers use it on the medical units; and (3) the relative costs and cost-effectiveness of the three different implementation strategies.

**Discussion:**

If implementation of Do One and Order One is successful, the field will have two alternative strategies for supporting medical providers’ proficient use of brief behavioral interventions, such as MI, for medical inpatients who use substances problematically.

**Trial registration:**

Clinical Trials.gov (NCT01825057)

## Background

Individuals who use substances are seven times more likely than non-substance-using individuals to be admitted to a hospital for medical care secondary to complications that are caused by their substance use [1]. Between 20 and 36 % of hospitalized patients are current smokers [2–4] and 10–40 % have an alcohol or drug use disorder [5–9], with high rates of co-occurrence among substance use disorders [2]. These rates far exceed those found in the general US population [10], demonstrating that inpatient hospitals provide care for a disproportionate share of patients who misuse substances [11]. These patients are costly to treat and at risk for poor health outcomes [12, 13]. Hospitalization provides a unique opportunity to identify and motivate patients to address their substance use problems in that patients are accessible, have time for an intervention, and are often admitted for complications related to substance use that renders hospitalization a “teachable moment” [14, 15].

### Motivational interviewing for substance use disorders

Motivational interviewing (MI) [16, 17] has been the basis of most brief behavioral interventions used in medical settings [18]. MI is a patient-centered approach that develops the patients’ motivation and commitment to change within a collaborative, highly empathic patient-clinician relationship. Clinicians blend a combination of fundamental patient-centered counseling skills (e.g., reflective listening) with advanced strategic methods (e.g., develop discrepancies between important life goals and substance use) to elicit and support patient statements that favor change (i.e., change talk) and decrease those against change (i.e., sustain talk). The aim is to have patients talk themselves into behavioral change.

MI has a strong evidence base in the treatment of substance use disorders (alcohol, drugs, nicotine), consistently demonstrating small to moderate clinically significant effects across targeted behaviors [19, 20]. As a brief intervention for primary care patients, MI has its most consistent support with non-dependent unhealthy alcohol use [21–25]. Some studies suggest that MI is more effective than expected with dependent drinkers in hospitals and other medical settings [26–28], as well as with younger adult, female, and alcohol-misusing medical inpatients [29]. MI also has promise for addressing medical patients’ illicit drug use [30, 31], though recent studies indicate that more evidence for its effectiveness in this arena is needed [32, 33]. In addition, MI’s key causal model of affecting patient language for and against change has been generally supported [34–38]. Miller and colleagues [17, 39] recommend the strength and frequency of in-session client change talk and sustain talk as good proxies for patient outcomes.

MI skills can be taught to a broad range of health care providers [40], with training gains that are medium to large [41, 42]. One-time workshop training has been the main strategy for teaching clinicians MI, especially in healthcare where formal continuing medical education (CME) events such as workshops are common [40, 43, 44]. MI workshop training alone consistently produces immediate increases in MI integrity (i.e., adherence and competence); however, without subsequent post-workshop training, these gains erode [41, 42, 45]. In contrast, when a workshop is followed by a competency-based supervision approach marked by direct observation of practice, MI integrity rating-based feedback, and coaching-specific MI practices, initial skill gains are sustained [41, 42].

### Implementing post-workshop feedback/coaching strategies within medical settings

Integration of substance use interventions into medical settings could improve health outcomes and reduce health care costs [24, 25, 31, 46, 47]. However, this is contingent upon effective implementation strategies. Implementation theories identify two important components for consideration when crafting strategies: complexity and compatibility. The more complex a strategy, the greater the difficulty in implementation [48–51]. Similarly, if a strategy is not compatible with the setting’s existing workflows and systems, implementation is likely to fail [48, 49, 51, 52]. In short, implementation strategies that are straightforward and fit into existing practices of medical providers and their workplaces are most likely to succeed [53].

Traditionally, an apprenticeship model has been used to instruct inpatient medical providers in bedside procedures [54–57], but this model has not been applied to promote the use of behavioral counseling techniques. Commonly referred to as “see one, do one,” the instructor explains the theory and techniques of a practice and demonstrates it in a simulated scenario or directly with patients. Subsequently, trainees practice the approach under the supervision of an expert provider who offers live performance feedback and coaching to improve the technique. This form of learning on the job has been a modus operandi in medical education for centuries [57] and is analogous to the competency-based supervision approach noted above. The ultimate aim of “see one, do one” is to have clinicians implement the procedure proficiently with their patients. One potential caveat to “see one, do one” is that it is somewhat complex because it requires appropriate patients and a trainer available for teaching. Moreover, when applied to behavioral counseling approaches like MI, it may be seen as incompatible with the clinicians’ medical role and time constraints [18].

Another common practice in general hospitals is the use of psychiatry consultation-liaison (CL) services. CL providers who are expert in a particular subspecialty of medicine, such as mental health and addiction problems [58], provide assessment and specialty guidance on the management of patients. Inpatient clinicians request a CL consult by ordering it through the electronic medical record, which is provided on the same day or day thereafter, depending on the urgency of the request. About 20 % of psychiatric CL consultations involve patients who have substance use problems [59–64]. The ultimate aim of CL is to have highly trained specialists implement the procedure proficiently with patients, rather than having the referring clinicians conduct it themselves. The use of CL as a promising vehicle for implementing specialized behavioral counseling approaches such as MI in inpatient medicine has never been tested. From the perspective of inpatient clinicians, using CL is a simple, minimally burdensome process (i.e., order one) and highly compatible with the way they secure other specialist services for their patients. The potential problems with this approach are as follows: (1) it requires clinicians to recognize and order the service; (2) patients must accede to a consultation with a substance abuse expert; (3) it may be more expensive since it requires additional staff time from individuals who have expert training and work on a specialty service; and (4) the treatment would be delivered by providers who are not central to the overall care of the patient, thus potentially reducing the potency of the intervention. Given the possible pros and cons of these approaches, we currently lack information about the most effective and cost-effective strategies by which to implement MI into a general inpatient medical setting.

### Study focus and aims

This study will examine the effectiveness of three different strategies for integrating MI into the practice of medical providers (nurses, physician assistants (PA), physicians) working within a general medical hospitalist service at a large, academically affiliated teaching hospital in Connecticut. We will randomize 30 providers to one of three conditions: (1) a continuing medical education workshop that “shows” providers how to conduct MI (the control condition, called See One); (2) a “see one, do one” apprenticeship model involving workshop training plus live supervision of bedside practice (Do One); and (3) ordering MI from CL after learning about it in a workshop (Order One). Following the respective MI trainings, each provider will be assessed for the provision of MI to 40 study-eligible inpatients, recruited by the research team after admission to the general medical units.

Our primary aims are to assess the uptake of MI by providers, the integrity by which they use MI, and the cost-effectiveness of the three implementation strategies. We hypothesize that the percentage of MI interviews in study-eligible inpatients per provider will be higher in both the Do One and Order One groups than the See One group. We also hypothesize that both the Do One and Order One groups will conduct sessions with greater MI adherence and competence than the See One group. Finally, we predict that See One will be the most cost-effective implementation strategy when the threshold monetary value to hospital decisionmakers is relatively low for more inpatients to receive an adequately conducted MI session, whereas Do One and Order One will be more cost-effective than See One when the threshold value is relatively high.

## Methods/Design

### Study design and overview

This study is a hybrid type 3 effectiveness-implementation trial [65] in that it primarily will evaluate the effectiveness of three different implementation strategies for integrating MI into a general medical hospitalist service, and secondarily examine proximal patient-level effects of MI in the form of in-session frequency and strength of patient change talk and sustain talk. Specifically, providers will be randomized to one of three conditions (See One, Do One, or Order One) and followed for their provision of MI to study-eligible/consented patients. Research staff also will recruit patients who are admitted to the general medical hospitalist service and assigned to a participating provider according to the hospital’s usual clinical administrative procedures. Thus, patients will follow the randomization condition of their provider, though providers will not know which patients on their caseloads have enrolled in the study. This approach will permit a naturalistic test of the providers’ ability to identify and intervene using MI with patients who have substance use problems. Each provider will be followed until he or she has cared for 40 study-enrolled patients, whether or not the provider has recognized the patient as a substance user and/or provided a MI intervention. Research staff will not tell the providers the target enrollment but rather will tell them when they have reached the “target” number and have completed the trial. In total, 1200 medical inpatients will be enrolled and may potentially receive a MI intervention. Post-trial, providers will participate in a qualitative interview that will determine implementation facilitators and barriers.

Primary outcomes will be (1) the percentage of MI sessions, as verified by audio recordings, conducted among each provider’s 40 consecutively enrolled study patients; (2) independently rated MI adherence and competence ratings of the sessions; and (3) the percentage of sessions conducted that meet a criterion level of adequate MI performance used in MI effectiveness [66–68] and clinician training trials [69, 70]. In addition, we will calculate the relative costs and cost-effectiveness of the three conditions. Secondary outcomes will be (1) independently rated strength and frequency of patient statements that favor (change talk) or disfavor change (sustain talk) in the sessions as a proxy for patient outcomes [17, 39], and (2) themes related to implementation facilitators and barriers identified through qualitative interviews.

### Setting

The proposed study is taking place on the general medical units of a university-affiliated teaching hospital. The general medical hospitalist service consists of PA and MD teams who share care of approximately eight medical inpatients daily. Providers typically see patients on more than one unit and see each assigned patient once or twice per day. The general internal medicine hospitalist teams cover about 160 beds/day (range = 130–210) on 13 different units, excluding intensive care units. In 2014, the service was responsible for approximately 10,000 discharges. The average length of stay for patients was 4 days. The psychiatric CL is available for all admitted patients. The physicians or PAs request CL services via a consultation order entered in the electronic medical record. Nurses can also identify the need for CL services; in these situations, the nurse contacts the physician or the PA on the team who places the order for CL services. Consultations are typically seen within 4 to 8 h of placement, depending upon the acuity and time sensitivity of the requested consult. Non-urgent consults may be seen the next day if the caseload is high. For the purposes of this study, a separate CL order and group of practitioners were put in place that would only offer the MI interview.

### Participants

Participants will be providers (physicians, PAs, nurses) and inpatients who are using tobacco or illicit drugs or misusing alcohol or prescription medications.

Inclusion criteria for provider participants are (1) assignment to one of the general medical inpatient units; and (2) agreement to all study procedures. Providers will be excluded if they (1) only work on intensive care units where patient morbidity would bypass interviewing; (2) have been formally supervised in MI in the past; or (3) intend to give notice to leave the hospital or are scheduled for medical or family leave during the study period.

Inclusion criteria for inpatient participants are as follows: (1) 18 years of age or older; (2) acknowledge use of drugs, alcohol, or nicotine within the past 28 days and meet screening criteria consistent with a substance use disorder; (3) have an expected length of stay of 2–3 days; and (4) willing to consent to audio recording of the MI session, should it occur. Patients will be excluded if they (1) have an altered mental status such as delirium, encephalopathy, dementia, or mental retardation that would impair provision of consent and ability to participate; (2) are unable to speak English; (3) are placed in an intensive care unit bed; (4) were previous study participants; and (5) have any other medical condition that investigators feel would make it too difficult to complete an assessment and MI interview (e.g., stroke, deafness, tracheostomy).

### Provider screening, recruitment, randomization, and reimbursement

The main unit of randomization will be the providers within the hospitalist service. The service includes physicians, PAs, and nurses who work on non-critical care general medical units. Prior to randomization, members of the study team will meet with the providers during staff meetings, wherein they will describe the project and elicit interest. Thereafter, interested providers will be screened for eligibility, give written informed consent for their participation, and complete baseline assessments. Following baseline assessments, a restricted randomization procedure generated by the study’s data manager will be used to allocate an equal number of providers to the three conditions (10 providers per condition); allocation will be concealed from all other research staff until condition assignment. Participants will be reimbursed $350 and eight CME credits for baseline assessments and workshop training and $50.00 for post-trial assessments.

### MI intervention

The practice under study for implementation is a single 20-min MI session used to train medical students and physicians [71, 72]. The MI session is based on the four main processes of MI [16]: (1) engage the patient to understand his/her substance use and motivations for change; (2) focus on the primary problematic substance; (3) evoke change talk and resolve sustain talk about the patient’s substance use; and (4) plan for change by developing a change plan or present change options for later consideration.

### Implementation conditions

Providers will each receive the MI implementation strategy offered within their assigned condition. Initially, providers will receive MI workshop training. They will be randomized to an implementation condition immediately upon completion of the workshop. In each implementation condition, all providers, as well as the CL clinicians in Order One, will audio record their MI interviews to confirm the interviews have occurred and to permit MI integrity rating.

#### Workshop only (See One)

Providers will participate in a 1-day skill-building workshop conducted by the first author (SM), a member of the Motivational Interviewing Network of Trainers (MINT), according to MINT recommendations, giving them an opportunity to “see” the MI intervention and learn how to conduct it through expert and video demonstrations and experiential activities. Providers will also be taught how to screen patients for risky substance use with the modified CAGE for alcohol and drugs [73] and Heaviness of Smoking Index [74].

#### Workshop plus live supervision (Do One)

Providers will participate in a 1-day skill-building workshop, as outlined above. Following the workshop, the providers will each “do one” MI intervention under the live “bedside” supervision of a CL clinician trained in MI. The study’s first author (SM) will have taught four CL clinicians MI and the accompanying supervisory practices developed in prior MI training work [69, 71, 72]. Providers will be supervised twice before beginning the trial and once midstream. In addition, they can request additional live supervision at any point during the trial, consistent with the apprenticeship model [54–57].

#### Workshop plus consultation-liaison service (Order One)

Providers will participate in a 1-day skill-building workshop, as outlined above. They will be instructed that they may either administer MI or “order” a MI interview to be delivered by a CL clinician with specialty MI training. Only providers in Order One may “order” a MI interview. We will have four CL clinicians available for ordered MI to provide adequate coverage.

Training CL clinicians in MI will follow a clinical trial training approach used in efficacy and effectiveness trials [75, 76]: (1) a 2-day skill-building workshop; (2) post-workshop supervised practice cases based on review of audio-recorded sessions; and (3) follow-up monthly group supervision to maintain and monitor the CL clinicians’ MI practice.

### Patient screening, recruitment, and reimbursement

Research staff will review a list of patients newly admitted to the general medical hospitalist service and assigned to one of the provider participants, approach patients not obviously excludable, explain the study procedures, and obtain verbal and written consent for screening. Patients who provide consent will complete a brief demographic questionnaire and be screened for (1) delirium; (2) alcohol, drug, or nicotine use within the past month; and (3) sufficient symptoms associated with their substance use. After screening eligibility is confirmed, patients will be consented and complete a computer intake that includes questions to self-identify the primary substance misused, addiction severity, depression symptoms, and functional health. Finally, they will be told that a provider may approach them on the unit to discuss their use of substances, which would be audio recorded as part of the study. Patient participants will be reimbursed $30 for their assessment, whether or not they ultimately receive MI.

### Procedures for monitoring audio recording of MI interventions

Providers will not be informed about which patients are consented by research staff. Providers must rely on usual procedures (e.g., notes in patients’ electronic medical records) and their workshop training to identify risky substance use and the need for MI. All providers will be issued a digital recording device. They will audio record their own MI sessions and give the recordings to research staff, along with identifying information that will enable the research staff to check the patients’ study enrollment status. If recordings are obtained from non-study participants, they will be immediately erased by research staff. Otherwise, they will be downloaded for storage on a secure server for later analysis. Research staff will send occasional messages to providers to remind them to record their MI interventions. As a back-up, additional recording devices will be conveniently placed on the units, and the administrative assistant on the CL service and research staff will have additional recording devices that can be given to providers on demand.

### Assessments

The proposed project will evaluate training effects using mixed methods to gather both quantitative and qualitative data [77, 78] and be organized according to the basic structure of Kirkpatrick’s four-level (reaction, learning, behavior, results) training evaluation model [79]. Only instruments central to our primary and secondary outcomes or that are less familiar are described in more detail. Provider assessments will occur at baseline, during the trial, and post-trial (i.e., after their 40th assigned study-enrolled patient has been discharged from the unit). Patient assessments will occur only at baseline.

#### Reaction level (reactions to implementation strategies)

The Workshop Evaluation Form and Supervision Evaluation Forms [69, 80] will evaluate the providers’ satisfaction with the workshops and supervision provided in the study. A Facilitators and Barriers Qualitative Interview will be used to assess the providers’ perception of the facilitators and barriers of screening patients for substance use, intervening with them, specifically using MI, and employing the different implementation strategies at baseline and post-trial. Focus group or individual interviews will be conducted with providers as they enter and exit the study. Key informant interviews will be conducted with the Director of Hospitalist Services, the Chief PA, and nurse unit managers to further assess organizational barriers and facilitators.

#### Learning level (changes in knowledge and attitudes)

Beliefs about MI [81] will assess the providers’ personal experiences with and beliefs about MI and perceived barriers to implementing it. The Motivational Interviewing Questionnaire [71] assesses the providers’ knowledge of MI principles. Clinician Rulers [71] assesses the providers’ interest, confidence, and commitment in using MI.

#### Behavior level (changes in behavior)

MI uptake will be based on the number of MI intervention sessions audio recorded by providers. The Independent Tape Rater Scale (ITRS) will assess the integrity of MI delivery and the criterion level of adequate MI performance within each session collected at baseline and during the trial. The ITRS includes items that cover therapeutic strategies that are MI consistent (e.g., reflections) or inconsistent (e.g., unsolicited advice). For each item, raters evaluate the practitioners for adherence (i.e., the extent of intervention delivery) and competence (i.e., the skill/quality of intervention delivery) along 7-point Likert scales. For our primary outcomes, we will (1) calculate mean adherence and competence scores for the two factors (fundamental and advanced MI strategies) identified in prior psychometric analyses [80, 82, 83]; and (2) determine if sessions achieve our criterion level for adequately performing MI [66–68], namely, at least half the MI consistent items rated average or above for both adherence and competence.

#### Result level (results that occurred from the application of the new practice)

Motivational Interviewing Skills Code 2.1 Client Language Coding System [84] will obtain frequency counts and strength indices of positive (change talk) and negative (sustain talk) language categories in four categories: reason (includes desire, ability, and need statements as subcategories); others (hypothetical advice to others, if-then statements about the possibility of changing, foretelling of future problems if change does not occur, problem recognition); taking steps; and commitment.

#### Provider, patient, and work environment characteristics

The Clinician Survey [85] will be used to collect baseline provider demographics and background. For patients, several instruments will be used: (1) Confusion Assessment Method—Shortened Version [86] to assess delirium symptoms seen at bedside; (2) Timeline Followback [87, 88] to assess patients’ past month self-reported substance use; (3) modified Mini-International Neuropsychiatric Inventory Clinician-Rated [89] to generate patient DSM 5 drug, alcohol, and nicotine use symptoms and diagnoses; (4) sections of the Addiction Severity Index [90] to assess the frequency, duration, and severity of substance use problems over the patients’ lifetime and in the past 30 days; (5) Patient Health Questionnaire—9 [91] to determine severity of depression; (6) Short Form Health Survey—12 [92] to measure patients’ functional health; (7) Medical Chart Review to obtain length of stay on the unit, admission and discharge diagnoses, confirmation of self-reported substance misuse (e.g., labs), and possible barriers to receipt of MI that may occur after consent or assessment (e.g., onset of delirium, cardiac arrest, early release from hospital); and (8) Motivation for Change Scale [93, 94], which uses three items (analog scale coded from 1 to 100) tapping patient drug, alcohol or tobacco use likelihood, problem recognition, and treatment motivation. Finally, the Nursing Work Index-Revised [95–97] was used, a 15-item scale to access provider perception of the hospitalist service work environment in terms of (1) autonomy in making patient care decisions; (2) control providers have over others to promote high-quality patient care; (3) collegiality with other medical staff; and (4) administrative/managerial support.

#### Cost estimates

We will estimate the costs of the three MI implementation strategies from the perspective of the provider (i.e., hospital) to increase the real-world usefulness of the cost estimates outside of this research protocol [98]. We will not include research costs (e.g., participant reimbursements) but rather restrict cost estimates to those associated with implementing the three MI implementation strategies. Our cost methodology will follow the micro-costing steps recommended by Yates [99] and Zarkin and colleagues [100]. We will first delineate relevant non-research activities (e.g., MI workshop training, MI interventions, supervision (including expert review of CL provider sessions)), and, for each identified activity, we will gather data on both the time spent by personnel in the activities and, as relevant, the space associated with each activity using a modified version of the Resource Allocation Worksheet developed for Project COMBINE [98]. This form will collect data on the total labor hours spent on each activity by the trainer, providers, and CL clinicians and the space used to conduct the activity. The labor costs of each activity will be equal to the product of the amount of time spent by each person on the activity and their fully loaded wage (i.e., including fringe and overhead). To estimate space costs, the research assistant will measure in square feet the size of the rooms used for training, MI interventions, and supervision. We will calculate an average space estimate per medical unit for the main activity domains (workshop training, MI intervention, supervision) and multiply these domains by the annual rent per square foot for the hospital. We will obtain salary data (actual wage plus fringe rate for salary staff and hourly contract rate for contract staff) for providers and CL clinicians and annual rent per square foot from administrators. We also will record all the direct material expenses (e.g., rating forms, recording devices) of conducting the MI training workshops and supervisions.

### Data analyses

#### Data analysis for quantitative primary and secondary outcomes

Descriptive statistics for all outcome variables will be calculated prior to statistical analysis. Continuous outcome variables will be evaluated for normality and transformations will be applied as necessary. Two-sided tests and overall alpha level of 0.05 for all primary hypotheses will be used.

The principal strategy for assessing the effectiveness of the study implementation conditions on outcome will be mixed effects general linear models for continuously measured primary (percentage MI sessions conducted, MI adherence, and competence) and secondary (strength and frequency of patient change and sustain talk) outcomes variables, and generalized linear mixed models for binary outcomes (e.g., meets criterion MI performance threshold). In both types of models, we will have training condition as the main predictor variable and will include random effects for providers to account for clustering of observations within providers. Our main hypotheses involve group comparisons with the See One group as the reference condition. We will consider significant comparisons of the Do One and Order One conditions to the See One condition in the predicted direction (e.g., more MI sessions conducted) as supportive of our hypotheses. Comparisons of Do One with Order One conditions will be conducted for exploratory purposes only.

#### Adequacy of sample size for primary hypotheses

Based on prior meta-analyses [41, 42, 101], we conservatively assume medium effect sizes for between-group comparisons. Intra-class correlations accounting for expected within clinician variance were estimated based on Imel et al. [102] and are expected to be small (in the 0.05 to 0.10 range). Feasibility constraints (i.e., 10 providers per condition) limited the number of provider clusters being considered. Finally, because we do not expect all providers to identify substance using patients and use MI with them (i.e., our first hypothesis about MI uptake), we will require a larger sample size of study-enrolled patients to reach our targeted number of audio-recorded MI sessions for hypothesis testing purposes. We estimate 80 % of patients across conditions will receive MI. Based on these estimates and constraints, alpha level of 0.05 and power of 80 %, 30 providers and 40 patients per provider will be sufficient for testing the primary hypotheses of the study in this cluster-randomized implementation trial. Table 1 shows the actual total sample size for the cluster-randomized design for continuous and binary outcomes, adjusted for a conservative estimate of MI uptake.

**Table 1:** Sample size estimates based on two-sided alpha = 0.05 for pairwise condition comparisons and 80 % power

Effect size	Total sample size for an ordinary RCT (3 arms)	ICC	Number of providers	Average number of patients per provider	Total sample size for a cluster RCT unadjusted for estimated MI uptake	Total sample size for a cluster RCT adjusted for estimated MI uptake
*d* = 0.4	300	0.07	31	31	930	1116
40 vs 18 %	195	0.1	30	17	510	765

#### Incremental cost-effectiveness analyses

The relative cost-effectiveness of the three MI implementation strategies will be assessed using both incremental cost-effectiveness ratios (ICERs) and cost-effectiveness acceptability curves (CEACs). Incremental cost-effectiveness analysis is the appropriate approach to use in this study inasmuch as Do One and Order One both add clear and certain costs to See One [103, 104]. ICERs and CEACs will be calculated from the provider’s (i.e., hospital) perspective. Using the cost estimates described in the Assessments subsection, we will calculate ICERs for multiple outcome measures, including (1) the number of MI sessions delivered and (2) the number of MI sessions delivered to criterion. The ICERs measure the incremental cost of using a given integration strategy, compared to the next-least-costly strategy, to produce an extra unit of effect for each of the outcomes. By using multiple outcomes, we can determine the robustness of our cost-effectiveness findings and provide a more fine-grained cost-effectiveness analysis to address different priorities (e.g., uptake of MI by providers on medical units, integrity of MI delivered to patients) that stakeholders may have [105, 106].

To illustrate the uncertainty associated with the ICER point estimates, costs and effects for each implementation strategy will be bootstrapped (with 2000 replicates) to produce confidence intervals around the ICERs and to produce CEACs for each of the outcome measures [107]. CEACs quantify the uncertainty in the cost-effectiveness analysis by showing the probability that each strategy is the most cost-effective for any given threshold value [107–109]. Finally, both scenario-based analysis (i.e., likely case, best case, worst case) and one-way sensitivity analysis will be conducted to determine the robustness of the cost-effectiveness results to alternative assumptions about a wide variety of implementation parameters (e.g., unit costs of labor, space, and materials). The results of the one-way sensitivity analysis will be presented graphically using a tornado diagram.

#### Qualitative data analyses

Each focus group and key informant interview will be audio recorded, transcribed, and independently coded using debriefing to discuss and challenge findings [110]. Grounded theory methods developed by Strauss and Corbin [111, 112] will be used to identify themes related to implementation facilitators and barriers across informants. The collection of data from multiple informants, iterative process of data collection and analysis, use of two researchers to code each transcript and work to consensus, keeping an audit trail of the data analysis process, and the theoretical sampling of themes and concepts will increase creditability, transferability, dependability, and confirmability of the findings [110]. We will identify the barriers and facilitators that are unique to and common across the conditions.

### Ethics

The study received approval from the Yale University Human Research Protection Program’s Human Investigation Committee and is registered at ClinicalTrials.gov (NCT01825057).

### Trial status

At the time of submission, the research team had recruited 30 clinicians and 813 patients. However, seven providers dropped out of the study before completing the trial (i.e., before reaching 40 opportunities to identify study patients and deliver MI) secondary to relocation, promotion, or re-assignment. These providers will be replaced to preserve the integrity of the randomized clustered study design, and as a result, recruitment will exceed the original targets of 30 providers and 1200 patients. Recruitment is ongoing and estimated to continue until January 2017.

## Discussion

This study will determine if two strategies commonly used to implement interventions with medical inpatients (Do One, Order One) are effective for integrating substance use treatment into general medical inpatient units, beyond the effects of traditional CME workshops (See One). The apprenticeship model represented by Do One is highly compatible with how medical providers learn new techniques following initial educational activities. However, medical providers may believe conducting behavioral interventions for substance use is outside their scope of practice, too difficult to perform well, or incompatible with their workflow and busy schedules. Alternatively, using CL allows them to “order” psychiatry staff to provide MI, making their task of providing critical addiction interventions to substance-using inpatients easier. However, if medical providers do not think addressing addictive behavior is central to their role, they may not actively screen patients for substance use or place an order for CL to deliver MI. Identifying how well each of these implementation strategies work, what factors facilitate or impede their use, and how much they cost will help inform future efforts to implement evidence-based addiction treatment services in medical hospitals.

### Limitations

The study is being conducted at only one large academically affiliated teaching hospital, which may limit the generalizability of its findings and disallow the examination of organizational differences (e.g., organizational readiness for change) that could influence the implementation outcomes [113]. Nonetheless, the study will assess the providers’ perception of their work environment (e.g., administrative/managerial support), which may point to some organizational factors that might affect MI uptake and its integrity. In addition, the study is not tracking patient substance use outcomes or measures of health care utilization (e.g., ER visits, rehospitalizations) and instead relies on proximal patient outcomes, namely, the frequency and strength of in-session patient change and sustain talk. Finally, this study only will follow the extent to which providers use MI during the trial and does not include a follow-up phase that assesses the sustainability of their practice. Should the Do One or Order One strategies prove to be more effective and cost-effective than See One, future studies should include a post-trial phase in which the sustainability of MI practice is measured.

## Conclusion

The profound cost and deleterious consequences of substance use dictate that evidence-based addiction treatments are made available to patients at all levels of the health care system. Inpatient medical hospitals serve as a catch bin for a large proportion of patients whose risky, hazardous, or disordered substance use drives or complicates their medical conditions. The extent to which different implementation strategies can effectively and cost-effectively help inpatient medical providers capitalize on this teachable moment requires careful scrutiny.

## Abbreviations

CEAC: cost-effectiveness acceptability curve
CL: consultation-liaison
CME: continuing medical education
ICER: incremental cost-effectiveness ratio
ITRS: Independent Tape Rater Scale
MI: motivational interviewing
MINT: Motivational Interviewing Network of Trainers
PA: physician assistant
